# Seroepidemiology and associated risk factors of brucellosis in small ruminants of district Khanewal, Pakistan

**DOI:** 10.5455/javar.2024.k741

**Published:** 2024-03-12

**Authors:** Abdul Sammad Ali Khan Shirwany, Mian Muhammad Awais, Muhammad Irfan Anwar, Muhammad Raza Hameed, Masood Akhtar, Nabeel Ijaz, Shakera Sadiq Gill, Muhammad Amjad Ali, Muhammad Sibtain Bhatti, Mamoona Chaudhry

**Affiliations:** 1 One Health Research Laboratory, Department of Pathobiology, Faculty of Veterinary Sciences, Bahauddin Zakariya University, Multan, Pakistan; 2 Department of Epidemiology and Public Health, University of Veterinary and Animal Sciences, Lahore, Pakistan; 3 Department of Clinical Sciences, Faculty of Veterinary Sciences, Bahauddin Zakariya University, Multan, Pakistan; 4 Livestock and Dairy Development Department, Directorate of Multan Division, Multan, Pakistan; † ASAKS and MMA contributed equally to this study and shared the first authorship.

**Keywords:** Brucellosis, small ruminants, seroprevalence, RBPT, indirect-ELISA, risk factors, Khanewal-Pakistan

## Abstract

**Objectives:**

Keeping in view the economic and veterinary public health importance of brucellosis, this research was conducted to determine its seroprevalence and associated risk determinants in small ruminants in district Khanewal, Southern Punjab, Pakistan.

**Materials and Methods:**

Two-stage cluster sampling technique was used for sampling, and the sample size was calculated using C-survey 2.0. Accordingly, sera samples (*n* = 392) were collected from small ruminants in the study area from October 2022 to July 2023. All the samples were tested for the presence of anti-*Brucella* antibodies by Rose Bengal Plate Test (RBPT), followed by confirmation of all the samples using an enzyme linked immunosorbent assay (ELISA) kit (ID.vet®, France; sensitivity and specificity=100%, each).

**Results:**

The seropositivity rate of brucellosis was 7.14% [*n* = 28/392; 95% confidence interval (CI) = 4.87%–10.12%] by RBPT, whereas the results of ELISA showed an overall seroprevalence rate of 7.40% (*n* = 29/392; 95% CI = 5.11%–10.37%) in the study population. Univariate analysis of risk factors revealed that abortion history (AH), retained fetal membranes (RFMs), repeat breeding, flock size (FS), educational status of farmers (ESFs), awareness about brucellosis (AB), and farm hygiene had a significant association with the seroprevalence of brucellosis (*p* < 0.05). The multivariate analysis using a binary logistic regression model revealed that variables including tehsil, FS, AH, RFM, ESF, AB, and farming system were significant factors (*p* < 0.05) associated with brucellosis in the target population.

**Conclusion:**

Brucellosis is prevalent in small ruminants in Khanewal, Pakistan. The disease burden can be reduced by improving the reproductive health of animals, farm hygiene, and farmers’ awareness about the diseases. Further studies are needed on a larger scale to devise stringent disease control strategies to avoid losses associated with brucellosis at regional, national, and global levels.

## Introduction

Brucellosis is an important but neglected bacterial disease with a significant impact on global health and the economy, especially in lower middle and lower income countries [1]. It affects a wide range of domesticated animals, including ruminants, pigs, and dogs, with zoonotic implications [2]. According to the World Health Organization and Food and Agriculture Organization, brucellosis is one of the most widespread zoonoses in the world, with an estimated 0.5 million cases annually [3]. Due to its zoonotic and public health importance, its control requires the coordinated efforts of stakeholders from the human and animal healthcare sectors for systematic surveillance to devise effective strategies [4]. It is caused by different species of *Brucella* (*B*.), which are Gram-negative cocco-bacilli, and its nine species are widely recognized*,* of which four species, including *Brucella melitensis, Brucella canis, Brucella abortus,* and *Brucella suis,* have been widely reported for zoonotic implications [5].

Apart from its public health importance, it also causes considerable economic losses in the livestock sector in terms of production losses, reproductive wastage, morbidity, medication, and veterinary costs, in addition to occasional mortality. It is mainly associated with late-term abortions in a wide range of animal hosts, but the most significant economic impact is due to the high cost of treatment [6]. Farm animals can acquire infection through a variety of routes, including licking the genitalia of sick animals and/or consuming water and food contaminated with the urogenital secretions of infected animals. *Brucellae* can also penetrate through skin and mucous membranes, and some species can be transmitted sexually through natural mating. A single infected male can sexually transmit the disease to several females [7]. A considerable variation in the prevalence of brucellosis (ranging from 1%–32%) has been reported in small ruminant populations of different parts of the world [8–10], and various socio-cultural and management/husbandry practices have been reported to contribute to this varying prevalence in small ruminants [4]. Control of brucellosis largely depends on intensive screening and surveillance of the illness, both in animals and humans. Various serological and molecular techniques are being used for its diagnosis, but culturing and isolating *Brucella* is still considered a gold standard method [11,12]. However, it is a risky and laborious method and requires sophisticated laboratory facilities in terms of biosafety to avoid any sort of biosecurity breach [13].

In Pakistan, the livestock sector contributes approximately 14.36% to the national Gross domestic product (GDP), supporting nearly 8 million families who rely on it for 35%–40% of their income. Small ruminants, being an integral part of this industry, are very common in rural areas with extensive or traditional farming systems [8,14], but this population is marked by a comparatively low growth rate and production in Pakistan. One of the major factors contributing to this low growth rate is the high endemicity of infectious diseases, with limited baseline data to devise and implement control measures. In this regard, a few studies have been conducted previously in different regions of the country regarding the prevalence and risk determinants of brucellosis [15,16], but the data regarding the status of this disease in small ruminants in South Punjab is scarce. Keeping this in mind, this research was conducted to ascertain the seroprevalence and associated risk factors of brucellosis in small ruminants in the district of Khanewal, Southern Punjab, Pakistan. The findings of this study will help in devising an effective strategy for the prevention and containment of disease in the region.

## Materials and Methods

### Study area and target population

**Figure 1.**
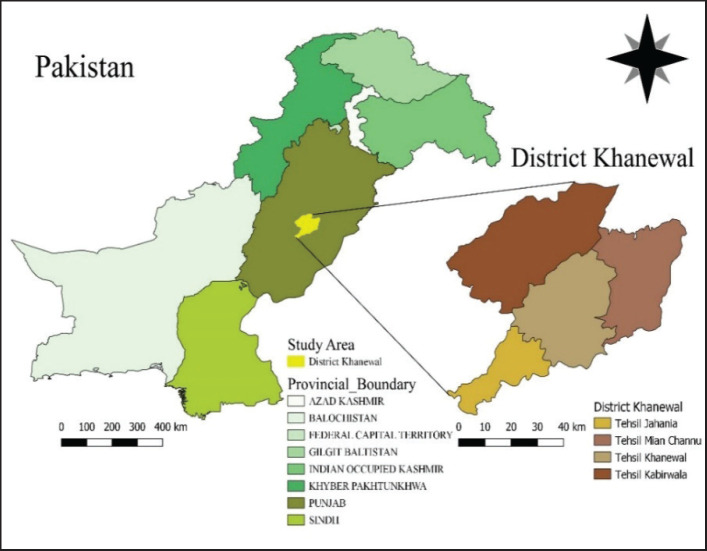
Map of the study area (developed using software QGIS; version 3.24.2).

This study was conducted from October 2022 to July 2023 to determine the seroprevalence and associated risk factors of brucellosis in small ruminants of district Khanewal, comprising four tehsils, including Khanewal, Kabirwala, Mian Channu, and Jahanian. It is situated at coordinates of 30°18′14.16″N, 71°55′47.57″E, with an area of 4,349 km^2^ (Fig. 1). The total population of small ruminants in the study area is approximately 718,129 heads [internal communication with the Livestock and Dairy Development Department, Govt. of Punjab; (L&DD) Department]. The average temperature of the study area is 31.79°C, with 2.79 km/h average windspeed, 23% average humidity, and 226 mm average rainfall annually. The district is crisscrossed by several canals and small rivers, which are used for irrigation purposes (retrieved from https://Khanewal.punjab.gov.pk, assessed on October 12, 2022).

### Ethical approval

Ethical approvals were obtained from the Institutional Animal Welfare and Ethics Committee of the Faculty of Veterinary Sciences, Bahauddin Zakariya University (FVS-BZU), Multan, Pakistan (No. FVS/AWEC-004/2020), and the institutional Ethical Review Committee of University of Veterinary and Animal Sciences, Lahore (No. DR/20). Prior consent was also obtained from the owners of small ruminants to utilize the data generated from this study for publication purposes without revealing their identity.

### Sampling technique

**Figure 2.**
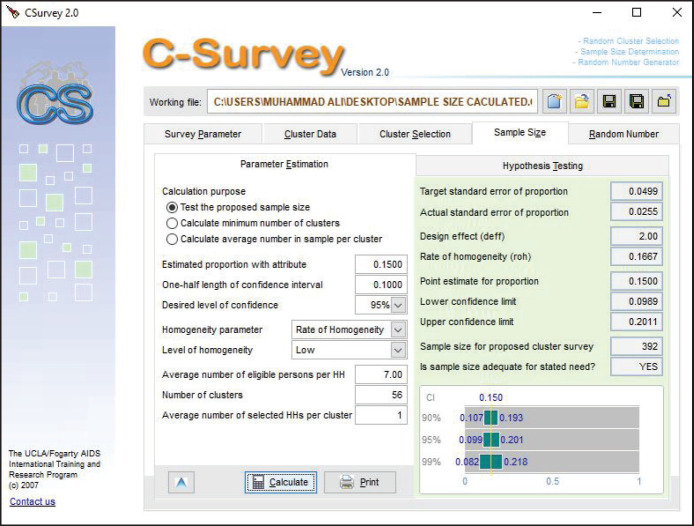
Sample size calculation of small ruminants using C-Survey (2.0)

A two-stage cluster sampling technique was used to select the study participants. To determine the sample size, software C-survey (2.0) was used, in which data on the small ruminant’s population across various villages in different tehsils of Khanewal district was entered as input, and the sample size, along with the number of clusters (villages) to be sampled and the average number of samples to be collected from each cluster was calculated. The software indicated a total sample size of 392, distributed across 56 clusters, and 7 samples from each cluster (Fig. 2).

The blood samples were collected from the target population with the help of L&DD Department. Approximately 3 ml of blood was aseptically collected from the jugular vein of each animal and shifted to pre-labeled gel clot activator tubes to harvest the sera samples. All the samples were transported to the One Health Research Laboratory, Department of Pathobiology, FVS-BZU, under optimal transport conditions and stored at −40°C until seroanalysis.

The descriptive epidemiological data regarding the pre-disposing factors (including age, breed, sex, body weight, abortion history (AH), physiological status, education of animal owners, parity, farm location, number of animals on a farm, medication history, vaccination schedule, and so on) were collected using well-designed questionnaires.

### Serological detection of brucellosis

All the sera samples underwent preliminary screening for brucellosis by commercially available Rose Bengal Plate Test (RBPT) antigen (VRI, Lahore, Pakistan), and samples showing agglutination within 3–4 min upon mixing with RBPT reagent at room temperature were considered positive. Positive and negative sera maintained at One Health Laboratory, FVS-BZU, were also used with each batch to validate the results.

Following RBPT, all the samples were subjected to a commercially available IDScreen brucellosis serum indirect multi-species diagnostic ELISA kit (ID.vet^®^, France; sensitivity and specificity = 100%, each). The assay was performed according to the instructions provided by the manufacturer. The validity of the kit and interpretation of results were done using IDSoft™ data-analysis software (Ver 5.11.6; ID.vet France).

### Statistical analysis

The data obtained from the analysis of the samples and questionnaires were further analyzed by univariate and multivariate statistical analysis using Minitab (version 19) and R-studio was the only interface that was used for R language. The differences were considered significant at *p* < 0.05. The inter rater reliability of the two serological tests was also determined by using Cohen’s kappa statistic. In univariate analysis, a total of 15 variables were analyzed for association with seroprevalence of brucellosis by the chi-square test, Fisher’s exact test, and odds ratio (OR). Whereas in multivariate analysis, all the risk factors were included in a backward elimination model that involved iteratively removing non-significant predictors to arrive at a more concise model that involved only significant risk determinants (*p* < 0.05). The significant variables in the backward elimination model were utilized to build the final binary logistic regression model (BLRM). This new model aimed to explore the relationships between the selected predictors and the positivity of brucellosis. The models were assessed using McFadden’s pseudo-*R*^2^ value and the area under the receiver operating characteristic curve (AUC-ROC).

## Results

### Overall seroprevalence of brucellosis

The results of RBPT showed a seropositivity rate of 7.14% [*n* = 28/392; 95% confidence interval (CI) = 4.87%, 10.12%], whereas iELISA showed an overall seroprevalence rate of 7.40% (*n* = 29/392; 95% CI = 5.11%, 10.37%) for brucellosis in the target population. The RBPT and ELISA tests showed almost perfect agreement with each other, with a kappa value of 0.83 by Cohen’s Kappa statistic.

### Univariate analysis of risk factors

The univariate analysis revealed that history of abortion (OR = 4.7; 95% CI = 2.05–10.91), retained fetal membranes (RFM) (OR = 3.5; 95% CI = 1.26–8.81), repeat breeding (OR = 3.68; 95% CI = 1.58–8.48), > 50 flock size (FS) (OR = 3.89; 95% CI = 1.48–10.26), uneducated farmers (OR = 3.59; 95% CI = 1.6–8.9), having awareness of brucellosis (OR = 0.149; 95% CI = 0.007–0.814), and poor farm hygiene (OR = 3; 95% CI = 1.2–9.25) had a significant association with seroprevalence of brucellosis in the target population (*p* < 0.05) (Table 1).

### Multivariate analysis of risk factors for brucellosis

**Figure 3.**
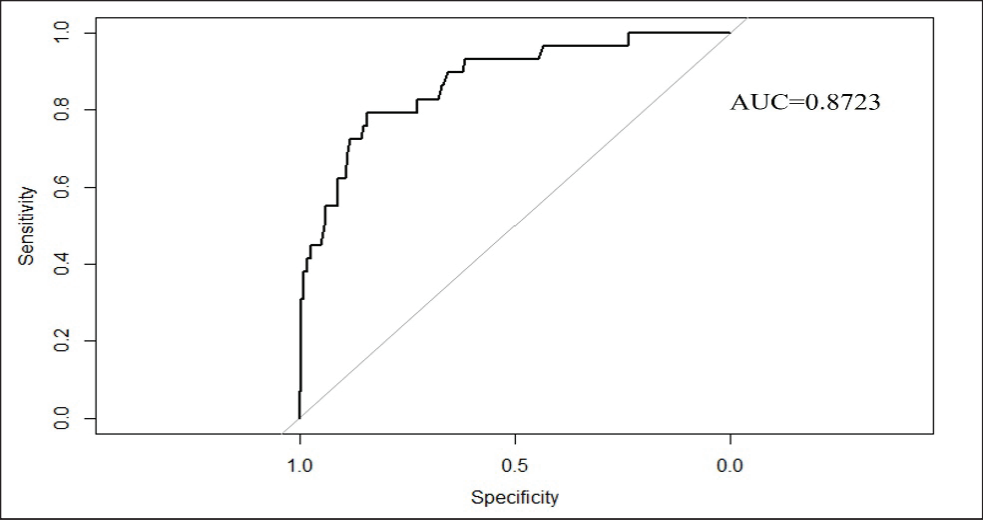
ROC curve of BLRM.

A total of 15 variables were selected for the backward elimination model, and based on the results, only 7 risk factors, *viz*., history of abortion, RFM, tehsil, FS, farming system, educational status, and awareness of farmers regarding brucellosis, were retained for the final BLRM model (Table 2). All the variables included in the final model were significantly (*p* < 0.05) associated with the seroprevalence of brucellosis in the study population (Table 3). The BLRM was assessed using McFadden’s pseudo-*R*^2^ value (*R*^2^ = 0.3222), and the AUC-ROC value of 0.8723 which indicated a good model fit (Fig. 3).

## Discussion

Brucellosis is an important but neglected zoonotic disease in most parts of the developing world, affecting both susceptible animal and human populations [1]. Unfortunately, epidemiological data on brucellosis is scarce in most parts of the country, including South Punjab, Pakistan, with a high population density of small ruminants.

Results of this study showed a higher seropositivity rate of brucellosis by iELISA (7.40%) as compared to RBPT (7.14%). This variation might be due to the longer diagnostic window and higher specificity and sensitivity of iELISA as compared to RBPT, and the same has been reported previously [17]. However, the literature also revealed that the stage of infection and the specific characteristics of the study population may also contribute to the variance [18]. Keeping in view the diagnostic capabilities of the two tests, it is recommended that RBPT be used as a cost-efficient preliminary screening tool, followed by confirmation through ELISA to ensure accurate and reliable results.

This study revealed that the overall seroprevalence of brucellosis in the small ruminant population of the district of Khanewal, Pakistan, was 7.40%, with an apparently higher prevalence in sheep (8.62%) as compared to the goat (6.12%) population. Some previous studies have reported an apparently lower prevalence of brucellosis in small ruminants of different parts of the world, such as 0.99% in the western borders of Pakistan [8], 6.6% in Kampala, Uganda [19], 5.59% in Tamil Nadu, India [20], and 5.8% in Kurdistan, Western Iran [21]. In contrast to current findings, some previous studies also reported a higher prevalence of brucellosis in small ruminants, such as 12.29% in district Kasur and Sheikhupura, Pakistan [15], 15.5% in goat and 8.6% in sheep populations of Baringo County, Kenya [22], 18.3% in Puducherry, India [10], 18.5% in Tarbiz, Iran [23], and 31.25% in goat and 22.5% in sheep populations of Matrouh governorate, Egypt [9]. The difference in values might be due to differences in diagnostic techniques, agroecological zones, geoclimatic conditions, husbandry practices, and traditions and culture [24].

**Table 1.** Univariate analysis of risk factors associated with brucellosis in small ruminants of Khanewal-Pakistan.

Variable	Variable level	Positives/Total	Seroprevalence (95% CI)	OR(95% CI)	Chi-square	*p*-value
Species-wise (RBPT)	Sheep	16/196	8.16 (4.92–12.08)	1.36 (0.62, 3.035)	0.615	0.433
	Goat	12/196	6.12 (3.38–10.29)	Ref.		
Species-wise (ELISA)	Sheep	17/196	8.67 (5.13–13.36)	1.45 (0.6736, 3.216)	0.931	0.335
	Goat	12/196	6.12 (3.38–10.29)	Ref.		
Tehsil-wise	Khanewal	6/126	4.76 (2.09–10.03)	0.42 (0.14, 1.22)	2.743	0.433
	Kabirwala	7/98	7.14 (3.23–13.96)	0.64 (0.23, 1.8)		
	Mian Chanu	7/84	8.33 (2.82–15.61)	0.76 (0.27, 2.14)		
	Jahanian	9/84	10.71 (5.50–19.31)	Ref.		
Age (Years)	≤3	13/213	6.10 (3.31–10.16)	Ref.	1.296	0.523
	>3 but ≤5	7/86	8.14 (3.67–15.92)	1.36 (0.52, 3.54)		
	>5	9/93	9.68 (4.95–17.43)	1.65 (0.68, 4)		
Weight (kg)	≤25	5/56	8.93 (3.58–19.18)	Ref.	0.258	0.7997*
	>25 but ≤40	15/216	6.94 (3.96–11.18)	0.76 (0.26, 2.19)		
	>40	9/120	7.50 (3.81–13.50)	0.83 (0.26, 2.59)		
Gender	Female	26/329	7.90 (5.36–11.30)	1.64 (0.55, 7.33)	0.761	0.597*
	Male	3/63	4.76 (1.30–12.98)	Ref.		
Number of parities	≤2	7/109	6.42 (2.90–12.55)	Ref.	1.233	0.54
	>2 but ≤4	9/124	7.26 (3.68–13.06)	1.14 (0.41,3.17)		
	>4	10/96	10.42 (5.34–17.95)	1.69 (0.62,4.64)		
Pregnancy	Yes	11/119	9.24 (4.88–15.72)	1.33 (0.57, 2.999)	0.461	0.497
	No	15/210	7.14 (4.07–11.50)	Ref.		
AH	Yes	14/74	18.92 (11.03–29.48)	4.7 (2.05, 10.91)	15.919	0.000
	No	12/255	4.71 (2.60–7.95)	Ref.		
Repeat breeding history	Yes	12/69	17.39 (9.65–27.99)	3.68 (1.58, 8.48)	10.8	0.001
	No	14/260	5.38 (3.12–8.72)	Ref.		
History of retention of fetal membranes	Yes	7/36	19.44 (8.83–35.75)	3.5 (1.26, 8.81)	7.399	0.014*
	No	19/293	6.48 (3.98–9.94)	Ref.		
FS	≤20	6/165	3.64 (1.59–7.66)	Ref.	8.775	0.012
	21 to 50	7/102	6.86 (3.10–13.41)	1.95 (0.64,5.98)		
	> 50	16/125	12.80 (7.72–19.79)	3.89 (1.48,10.26)		
ESF	Uneducated	21/173	12.14 (7.89–17.77)	3.59 (1.6, 8.9)	10.159	0.001
	Educated	8/219	3.65 (1.62–6.92)	Ref.		
Awareness of brucellosis in farmers	Yes	1/71	1.41 (0.07–7.25)	0.1494 (0.007, 0.814)	4.54	0.033
	No	18/321	8.72 (5.95–12.36)	Ref.		
Hygienic condition	Poor	24/245	9.80 (6.49–14.15)	3 (1.2, 9.25)	5.484	0.019
	Good	5/147	3.40 (1.34–7.58)	Ref.		
Farming system	Sheep farming system	5/99	5.05 (2.01–11.26)	0.54 (0.17,1.71)	4.689	0.204*
	Goat farming system	3/82	3.66 (1.00–9.96)	0.38 (0.1,1.5)		
	Both sheep and goat	8/89	8.99 (4.00–16.55)	Ref.		
	Mixed small and large ruminant	13/122	10.66 (5.80–17.38)	1.21 (0.48,3.05)		

*Fisher’s exact test *p*-value was applied when 1 or more observations had an expected count less than 5.

Ref = Reference.

**Table 2.** Multivariate analysis of risk factors associated with brucellosis based on backward elimination model on all variables.

Variable	Variable level	Regression coefficient	Standard error	Adjusted OR	95%CI OR		*Z*-value	*p*-value
					Lower	Upper		
Tehsil	Kabirwala	−0.8349	0.7777	0.43	0.09	1.99	−1.0735	0.283
	Khanewal	−2.8041	0.7926	0.06	0.01	0.29	−3.5380	<0.001
	Mian Channu	−1.2236	0.7036	0.29	0.07	1.17	−1.7391	0.082
	Jahanian	Ref.						
FS	≤20	−2.1057	0.6504	0.12	0.03	0.44	−3.2375	0.001
	21 to 50	−0.9809	0.5635	0.37	0.12	1.13	−1.7406	0.081
	> 50	Ref.						
Farming system	Goat farming system	−1.5241	0.9282	0.22	0.04	1.34	−1.6420	0.100
	Mixed small and large ruminant	0.4134	0.6073	1.51	0.46	4.97	0.6807	0.496
	Sheep farming system	−2.2124	0.8522	0.11	0.02	0.58	−2.5961	0.009
	Both sheep and goat	Ref.						
AH	Yes	1.2777	0.5464	3.59	1.23	10.47	2.3385	0.019
	No	Ref.						
Retention of fetal membranes history	Yes	2.2437	0.7234	9.43	2.28	38.92	3.1015	0.001
	No	Ref.						
AB	Yes	−2.6138	1.1998	0.07	0.01	0.77	−2.1785	0.029
	No	Ref.						
ESF	Uneducated	1.7401	0.5358	5.70	1.99	16.28	3.2477	0.001
	Educated	Ref.						

Ref, Reference

McFadden’s pseudo-*R*^2^ = 0.3566; AIC = 165.07.

Similar to current findings, some previous studies also reported a non-significantly higher prevalence of brucellosis in the female population of small ruminants as compared to males [16,20]. A slightly higher prevalence in females might be correlated with the chances of an infectious agent staying for a longer time within the female reproductive tract, providing a potential reservoir for the organism to proliferate. In addition, the presence of erythritol sugar in the placenta might also support the proliferation of *Brucella* in the gravid uterus, rendering them more susceptible to brucellosis [25,26].

In the current study, greater FS (> 50 heads) of small ruminants was shown to be significantly associated with brucellosis by both univariate and multivariate analyses. Previously, Gompo et al. [27] and Sorsa et al. [28] also reported similar findings. This association can be attributed to increased transmission opportunities in more extensive flocks due to the closer proximity of animals. In addition, management and implementing biosecurity practices can be more challenging in larger flocks, potentially resulting in reduced control measures. Moreover, in the current study, a protective effect was observed in exclusive sheep (OR = 0.22) or goat (OR = 0.15) farming systems, in comparison to other farming systems, that could be attributed to cross-species transmission of *Brucella*. Hussen et al. [29] also reported similar findings in their study conducted on a small ruminant population in eastern Ethiopia. It might be attributed to more effective biosecurity measures and lower interaction between different livestock species in exclusive farming systems. Additionally, specific management practices within these systems might contribute to minimizing the risk of disease transmission.

**Table 3.** Multivariate analysis of risk factors associated with brucellosis based on logistic regression on the significant variables selected by backward elimination.

Variable	Variable level	Regression coefficient	Standard error	Adjusted OR	95%CI OR		*Z*-value	*p*-value
					Lower	Upper		
Tehsil	Kabirwala	−0.8349	0.7777	0.53	0.13	2.14	−1.0735	0.283
	Khanewal	−2.8041	0.7926	0.05^a^	0.01	0.24	−3.5380	0.000
	Mian Channu	−1.2236	0.7036	0.39	0.10	1.47	−1.7391	0.082
	Jahanian	Ref.						
FS	≤20	−2.1057	0.6504	0.12^b^	0.04	0.42	−3.2375	0.001
	21 to 50	−0.9809	0.5635	0.42	0.14	1.21	−1.7406	0.082
	> 50	Ref.						
Farming system	Goat farming system	−1.5241	0.9282	0.15	0.03	0.79	−1.6420	0.101
	Mixed small and large ruminant	0.4134	0.6073	1.14	0.37	3.54	0.6807	0.496
	Sheep farming system	−2.2124	0.8522	0.22^c^	0.05	0.94	−2.5961	0.009
	Both sheep and goat	Ref.						
AH	Yes	1.2777	0.5464	3.64^d^	1.33	9.97	2.3385	0.019
	No	Ref.						
Retention of fetal membranes history	Yes	2.2437	0.7234	9.98^e^	2.48	40.13	3.1015	0.002
	No	Ref.						
AB	Yes	−2.6138	1.1998	0.10^f^	0.01	0.99	−2.1785	0.029
	No	Ref.						
ESF	Uneducated	1.7401	0.5358	6.16^g^	2.18	17.43	3.2477	0.001
	Educated	Ref.						

^a^The odds of testing positive for brucellosis were 0.05 (95% CI = 0.01–0.24) times lower in tehsil Khanewal compared to tehsil Jahanian.

^b^Smaller FS reduced the odds of brucellosis by a factor of 0.12 (95% CI = 0.04–0.12).

^c^In exclusive sheep farming, small ruminants exhibited protective odds (0.22; 95% CI = 0.05–0.94) of brucellosis as compared to both sheep and goat farming system.

^d^A history of abortion increased the odds of testing positive with brucellosis by 3.64 (95% CI = 1.33–9.97) times.

^e^The odds of retention of fetal membranes were 9.98 (95% CI = 2.48–40.13) times higher in brucellosis-positive small ruminants.

^f^Farmers being AB decreased the odds of brucellosis by a factor of 0.1 (95% CI = 0.01–0.99).

^g^Farmers who were uneducated increased the odds of brucellosis by 6.16 (95% CI = 2.18–17.43) times.

Ref, Reference.

McFadden’s pseudo-*R*^2^ = 0.3222; AIC = 166.19.

Reproductive disorders such as orchitis, abortion, RFM, repeat breeding, and sterility are frequently reported symptoms of brucellosis [4,6]. Results of the current study revealed that a history of reproductive disorders, including abortion, and RFM had a significant association with the seroprevalence of brucellosis in female small ruminants (*p* < 0.05). Similar findings have been reported in some previous studies [30–32]. Contrarily, some previous studies had also reported a non-significant association between brucellosis and a history of reproductive disorders [20,33]. The varied results in this study might be due to various factors, such as differences in animal raising patterns in the study area, an insufficient disease surveillance system, and poor management practices like the introduction of new animals into flocks without proper screening and quarantine procedures, coupled with the inability to cull infected animal heads [34].

A significant association was revealed between brucellosis in small ruminants and the educational status and awareness of their farmers regarding brucellosis. The results of the current study are also in agreement with the findings of previous studies [35,36]. The increased seroprevalence of brucellosis among farmers with low educational status and a lack of awareness regarding brucellosis can be attributed to limited knowledge about the dynamics of disease transmission and preventive measures, leading to poor hygiene practices and a lack of initial and regular screening of animals. The higher seroprevalence of brucellosis in small ruminants owned by illiterate farmers might also be due to traditional farming practices and socioeconomic challenges, in addition to limited access to veterinary services and vaccination.

Univariate analysis indicated a significant association between hygienic conditions and the seroprevalence of brucellosis in small ruminants. However, this risk factor was computed to be non-significant by multivariate analysis. Previous studies [37,38] also reported a significant association between farm hygiene practices (FHPs) and the prevalence of brucellosis. The increased seroprevalence of brucellosis in small ruminants in cases of poor FHP might be due to several factors, as poor hygiene can lead to direct contact between infected and susceptible animals, increasing the likelihood of transmission. In addition, improper cleaning and disinfection of animal housing and feeding areas can create a contaminated environment where *Brucella* can persist, and inadequate sanitation may facilitate its transmission among animals. Furthermore, the improper disposal of animal waste and aborted material may also contribute to the spread of the disease [39].

## Conclusion

In conclusion, brucellosis is prevalent in small ruminants in the district of Khanewal, Pakistan. The risk factors, including location/tehsil, history of abortion, retention of fetal membranes, FS, educational status of farmers (ESFs), awareness about brucellosis (AB), and farming systems, had a significant association with the seroprevalence of brucellosis in the small ruminant population of the study area. Effective control of these identified risk factors might decrease the incidence of brucellosis in the study area. It is highly recommended to formulate and implement prevention and control strategies with a major emphasis on inculcating AB in small ruminant farmers for the containment of infection in the region. Further studies are required to better comprehend the transmission dynamics and distribution of brucellosis at provincial and national levels. It will provide evidence-based data to animal health policymakers for the formulation of region-specific control strategies, allocation of resources, and collaborative efforts among relevant stakeholders.

## List of abbreviations

AB, Awareness about brucellosis; AH, Abortion history; AUC-ROC, Area under the receiver operating characteristic curve; *B*, *Brucella*; BLRM, Binary logistic regression model; CI, Confidence interval; ELISA, Enzyme linked immunosorbent assay; ESF, Educational status of farmers; FHP, farm hygiene-practices; FS, Flock size; FVS-BZU, Faculty of Veterinary Sciences, Bahauddin Zakariya University; L&DD, Livestock and Dairy Development Department, Govt. of the Punjab; OR, Odds ratio; RBPT, Rose Bengal Plate Test; Ref, Reference; RFM, Retention of fetal membranes.
